# RTK–RAS pathway alterations in hepatocellular carcinoma and cholangiocarcinoma: a multicohort genomic and single-cell transcriptomic study

**DOI:** 10.3389/fonc.2026.1963032

**Published:** 2026-10-01

**Authors:** Chongyuan Chen, Rongchun Xing, Yuhong Wen, Meiqi Li, Mingzheng Hu

**Affiliations:** 1 The First College of Clinical Medical Science, China Three Gorges University, Yichang, Hubei, China; 2 Department of Hepatobiliary Surgery, Yichang Central People’s Hospital, Yichang, Hubei, China

**Keywords:** genomic testing, hepatocellular carcinoma, intrahepatic cholangiocarcinoma, MAPK, RTK-RAS pathway, single-cell RNA sequencing

## Abstract

**Objective:**

To determine whether heterogeneous genomic alterations detected by clinical tumor panels converge on a reproducible pathway-level difference between hepatocellular carcinoma (HCC) and cholangiocarcinoma (CCA), and to examine its transcriptional output, cellular localization, and tumor–stromal coexpression context in public multi-omics cohorts.

**Methods:**

The institutional cohort comprised 52 patients who underwent tumor tissue genomic testing, including 28 with HCC and 24 in a CCA working cohort whose intrahepatic location was not uniformly documented. Ten canonical oncogenic pathways were compared using panel-aware evaluable denominators, and the selected pathway was tested in independent Memorial Sloan Kettering (MSK) cohorts. Matched genomic and transcriptomic data from The Cancer Genome Atlas (TCGA) were used to evaluate downstream pathway activity. GSE151530 and GSE189903 were analyzed by patient-level pseudobulk to localize the transcriptional difference, followed by CellChatDB-guided ligand–receptor coexpression analysis.

**Results:**

Receptor tyrosine kinase–RAS (RTK–RAS) showed the strongest cancer-type difference in the institutional cohort: alterations were detected in 4/21 evaluable HCC cases and 8/11 evaluable CCA cases (odds ratio [OR] = 9.44; 95% CI, 1.87–47.70; P = 0.00601; Benjamini–Hochberg-adjusted q = 0.0601), with concordant directions across sensitivity analyses. In MSK, integrated RTK–RAS alterations occurred in 240/412 intrahepatic cholangiocarcinoma (iCCA) cases (58.3%) and 107/289 HCC cases (37.0%; OR = 2.37; 95% CI, 1.74–3.22; P = 3.12 × 10^−8^). In TCGA, RTK–RAS status was associated with higher mitogen-activated protein kinase (MAPK) activity in the pooled cancer-type-adjusted model (β = 0.240; 95% CI, 0.051–0.428; P = 0.0129), whereas the within-iCCA estimate was positive but imprecise. Patient-level single-cell meta-analysis localized the higher iCCA MAPK activity predominantly to malignant cells (pooled effect = 1.455; 95% CI, 0.658–2.252; P = 3.46 × 10^−4^; q = 0.00208). Platelet-derived growth factor (PDGF)–PDGFRA and insulin-like growth factor (IGF)–IGF1R coexpression patterns accompanied this malignant-cell phenotype.

**Conclusion:**

Panel-aware integration of clinical genomic testing and public multi-omics data supports a higher burden of heterogeneous RTK–RAS alterations in CCA/iCCA than in HCC and links this difference to a malignant-cell MAPK transcriptional phenotype. The pathway-level framework complements, rather than replaces, established alteration-specific interpretation and generates tumor–stromal coexpression hypotheses for further spatial and functional testing.

## Introduction

Hepatocellular carcinoma (HCC) and intrahepatic cholangiocarcinoma (iCCA) are two major histologic types of primary liver cancer, with distinct cellular lineages, morphologic features, and clinical management paradigms (1–5). Nevertheless, genomic and transcriptomic studies indicate that the two diseases are not separated by an absolute molecular boundary. Some driver genes and molecular subclasses overlap, while each tumor type retains preferential oncogenic signaling programs (6–8). The distinction between HCC and iCCA may therefore extend beyond differences in the frequencies of a small number of mutations and involve systematic differences in how oncogenic pathways are perturbed.

Previous studies have established the major genomic differences between HCC and iCCA. TP53 alterations, WNT/β-catenin dysregulation, and cell-cycle abnormalities are frequent in HCC, whereas FGFR2 rearrangements, IDH1/2 mutations, and alterations involving KRAS and BRAF are enriched in iCCA (9–21). Several of these events already have direct clinical relevance, including FGFR2 fusions/rearrangements and BRAF V600E (22–26). The unresolved issue is therefore not whether these individual genes differ between the diseases. Routine clinical panels report point mutations, copy-number alterations, and fusions or rearrangements as separate events, and their evaluable denominators vary with panel content. A gene-by-gene comparison can consequently fragment biologically related inputs that converge on the same signaling pathway. Fixed pathway definitions combined with panel-aware denominators offer a way to evaluate that convergence without treating untested genes as negative (27, 28).

A pathway-level genomic difference is biologically more informative when it is accompanied by a corresponding downstream signaling output. Proteogenomic studies show that distinct driver events can produce related but non-identical signaling states, while single-cell studies reveal marked variation among malignant cells and within the iCCA microenvironment (29–34). A cross-cohort framework is therefore needed to ask whether heterogeneous genomic inputs converge on a reproducible transcriptional phenotype, where that phenotype is located at single-cell resolution, and what stromal expression context accompanies it.

Using genomic testing reports generated in routine clinical practice, we first compared 10 fixed oncogenic pathways between HCC and a cholangiocarcinoma (CCA) working cohort under explicit assay-coverage rules. We did not seek to rediscover established single-gene biomarkers. Instead, we asked whether one pathway-level difference would emerge from the common screening framework, replicate in an independent genomic cohort, correspond to its expected transcriptional output, and localize to a defined cellular compartment. Bulk and single-cell transcriptomic data were then used to examine the downstream mitogen-activated protein kinase (MAPK) phenotype and its associated tumor–stromal ligand–receptor coexpression context.

## Methods

### Patient inclusion and genomic report curation

The institutional cohort comprised patients who underwent tumor tissue sampling at Yichang Central People’s Hospital between June 2022 and September 2024 and whose tissue specimens were submitted to Shanghai 3D Medicines Medical Laboratory for genomic testing. The tumor type recorded in the testing report was either “liver cancer” or contained the term “bile duct.” Because the reports did not consistently distinguish intrahepatic, perihilar, and distal cholangiocarcinoma, all institutional biliary cases are termed the CCA working cohort; the term iCCA is reserved for public cohorts with explicit intrahepatic classification. Six institutional reports explicitly stated iCCA, and hepatic sampling supported an intrahepatic origin in five additional cases; these 11 cases formed a prespecified case-definition sensitivity set. All patients were deidentified and assigned a unique study identifier. The primary genomic report, supplementary files, and programmed death-ligand 1 (PD-L1) report for the same patient were linked by study identifier; file checksums were used for deduplication and completeness verification. PD-L1 reports were used only for file-completeness auditing and were not included in the genomic comparison. The testing platforms comprised the GK, GK-E, bht-E, and 35-gene pcp panels. The primary analysis was restricted to the broad GK, GK-E, and bht-E panels because they provided coverage of the 10 prespecified oncogenic pathways. The pcp panel was excluded from primary pathway screening because of its narrower coverage and unequal use between groups. Patient characteristics, specimen type, testing panel, report-positive genes, and alteration classes detectable by each panel were structured from the original reports and checked against the source PDFs. SHA-256 checksums were retained for files and analytic inputs. Reporting followed the Strengthening the Reporting of Observational Studies in Epidemiology (STROBE) principles (35).

### Assay coverage and definitions of gene- and pathway-level alterations

A gene-level alteration was defined as a positive event for that gene recorded in the clinical testing report. For each patient–gene pair, an unreported event was classified as negative only when the panel used for that patient covered the prespecified alteration class required for the gene; otherwise, the pair was considered not evaluable. Gene-level comparisons required at least 10 evaluable patients in each of the HCC and CCA working cohorts and at least two positive events across both groups combined.

Pathway-level analyses used the fixed membership of the 10 canonical oncogenic pathways defined by Sanchez-Vega et al. from The Cancer Genome Atlas (TCGA): Cell Cycle, Hippo, MYC, Notch, NRF2, PI3K, receptor tyrosine kinase–RAS (RTK–RAS), TGF-β, TP53, and WNT (27). Pathway members were not added or removed in response to findings in the institutional cohort. A patient was classified as pathway-altered when at least one report-positive event was present in any member gene. A pathway was classified as unaltered only when the required genes and alteration classes were evaluable and no positive event was detected.

### Comparison of pathway alterations between HCC and CCA and candidate-pathway selection

In the primary institutional analysis, alteration proportions were compared between the HCC and CCA working cohorts separately within the gene-level and pathway-level testing families. Two-sided Fisher’s exact tests were used for between-group comparisons. To retain an estimable effect size in the presence of zero cells, 0.5 was added to each cell of the 2 × 2 table and Haldane–Anscombe-corrected odds ratios (ORs) with 95% confidence intervals (CIs) were calculated. The Benjamini–Hochberg (BH) procedure was applied separately to the gene and pathway families to control the false discovery rate (36).

A candidate pathway in the discovery stage was required to meet all of the following criteria: pathway-family BH-adjusted q ≤ 0.10, at least four positive events across both groups combined, and a consistent direction of effect in the prespecified sensitivity analyses. Sensitivity analyses included all panels with evaluable denominators defined by actual coverage, restriction to CCA cases with report-based support for an intrahepatic origin, restriction to surgical specimens, and sequential exclusion of each broad panel. Pathways satisfying these criteria were ranked by q value, effect size, and event count, and only one candidate was advanced to external validation. Gene-level results were used to describe the composition of the candidate pathway and were not used to replace the pathway candidate *post hoc*.

### External validation of the RTK–RAS pathway in independent MSK cohorts

External validation data from Memorial Sloan Kettering Cancer Center (MSK) cohorts were obtained through the public cBioPortal interface (37, 38). The iCCA cases were derived from the ihch_msk_2021 study, for which the cohort and sequencing methods were reported by Boerner et al.; the HCC cases were obtained from the public hcc_msk_2024 study, whose hepatobiliary genomic cohort corresponds to the report by Song et al. (39, 40) Eligible patients had an unambiguous clinical tumor type, a primary solid-tumor specimen, and available mutation, discrete copy-number, and structural-variant data. One sample was retained per patient. If multiple samples were available, the representative sample was selected sequentially by prioritizing a primary lesion, an earlier sampling date, and then the lexicographically first sample identifier.

The 341-, 410-, 468-, and 505-gene versions of MSK-IMPACT (Integrated Mutation Profiling of Actionable Cancer Targets) jointly covered 38 genes in the fixed RTK–RAS pathway; ERF, ERRFI1, GRB2, and SOS1 were not covered by all four versions. The external primary analysis therefore used the same 38-gene common-coverage core in both cancer types (41, 42). Eligible events were non-synonymous somatic single-nucleotide variants or insertions/deletions, discrete copy-number values of +2 or −2, and structural variants with at least one breakpoint involving a target gene. Structural variants were retained as panel-detected events reported in the public cBioPortal files. Records explicitly labeled “Archer” in the event annotation were tabulated separately; the absence of such a label was not interpreted as orthogonal confirmation. Uniform fluorescence *in situ* hybridization (FISH) or RNA-based confirmation was not available in the public files (43). A patient with at least one eligible event was classified as positive for a core RTK–RAS alteration.

The primary external-validation endpoint was the patient-level prevalence of integrated RTK–RAS core alterations in iCCA versus HCC. The primary comparison used a two-sided Fisher’s exact test and reported the OR and 95% CI. To assess the influence of panel-version composition, effects were estimated separately within the IMPACT341, IMPACT410, and IMPACT468 strata, and a panel-stratified common OR was calculated using the Cochran–Mantel–Haenszel method. Prespecified supplementary analyses evaluated non-synonymous mutations, mutations plus deep copy-number alterations, and structural variants separately.

### Association between RTK–RAS alterations and MAPK pathway activity in TCGA

The TCGA analysis included primary HCC tumors from TCGA-LIHC and primary iCCA tumors from TCGA-CHOL with either a clinical designation of “Intrahepatic Cholangiocarcinoma” or an OncoTree code of “IHCH.” (7, 8) One primary tumor sample was retained per patient, and only cases with RNA expression, somatic mutation, discrete copy-number, and structural-variant data were included.

RTK–RAS genomic status in TCGA was defined using the complete 42-gene set and the same eligible event classes as in the MSK analysis. RNA-Seq by Expectation-Maximization (RSEM) expression values were transformed as log2(RSEM + 1). Pathway activity was inferred from downstream response genes using the human PROGENy model with top = 500 and scale = TRUE; all 14 pathways available in the model were calculated in a single run (44). For each pathway, the following linear model was fitted:


$$\text{pathway score}=\beta_{0}+\beta_{1}\left(\text{RTK}-\text{RAS status}\right)+\beta_{2}\left(\text{cancer type}\right)+\epsilon .$$

The coefficient β_1_ represented the difference in pathway activity between RTK–RAS-altered and unaltered tumors after adjustment for cancer type. MAPK was the prespecified primary transcriptional endpoint, and the effects of RTK–RAS status across the 14 pathways were adjusted using the BH procedure. Robustness analyses comprised cancer-type-specific models for HCC and iCCA, mutation-plus-copy-number status, mutation-only status, 10,000 within-cancer-type permutations of alteration-status labels, and 2,000 patient-level bootstrap resamples stratified by cancer type.

### Single-cell RNA-sequencing data processing

Single-cell analyses used two public liver cancer cohorts, GSE151530 and GSE189903 (30, 31). The retained six-compartment input from GSE151530 comprised 25 patients with HCC and 12 with iCCA; after cell-type-specific quality control, the malignant-cell analysis included 16 HCC and nine iCCA patients. GSE189903 comprised four HCC and three iCCA patients with multiple tumor cores, tumor margins, and adjacent non-tumor regions; its malignant-cell analysis retained four HCC and two iCCA patients. Cell-type annotations from the source studies were consolidated into malignant cells, T/natural killer (NK) cells, B cells, tumor-associated macrophages (TAMs), cancer-associated fibroblasts (CAFs), and tumor endothelial cells (TECs). Adjacent non-tumor regions were excluded. GSE125449 was not treated as an additional independent cohort because all 19 of its HCC/iCCA patient identifiers were already represented in the GSE151530 input; adding it would duplicate biological replicates (45).

To avoid treating cells or regions from one patient as independent biological replicates, raw counts were summed within each sample-by-cell-type combination to create pseudobulk matrices, requiring at least five cells per combination. Pseudobulk counts were normalized by the trimmed mean of M values (TMM) in edgeR and transformed to log counts per million (logCPM; prior count = 2) (46–48). In GSE189903, region-level scores were calculated first and then averaged within each patient before any between-disease model was fitted. All inferential comparisons used the patient as the independent statistical unit, consistent with recommended approaches for controlling pseudoreplication in multisample single-cell studies (49–51).

Within each dataset and cell type, activities of all 14 PROGENy pathways were calculated and a linear model estimated the patient-level iCCA-minus-HCC effect (52). Dataset-specific effects were combined by inverse-variance fixed-effect meta-analysis; normalized inverse-variance weights, Cochran’s Q, and I^2^ were reported. MAPK comparisons across six cell types were adjusted by the BH procedure, and a separate global BH correction was applied to all pathway-by-cell-type combinations. The malignant-cell Uniform Manifold Approximation and Projection (UMAP) was used only for visualization; cells were not treated as independent observations in cancer-type comparisons.

### CellChatDB-guided ligand–receptor coexpression analysis

Ligand–receptor priors were obtained from the human CellChatDB (53). Twenty-one receptor genes were selected from the fixed RTK–RAS set, and interactions in the “Secreted Signaling” category were retained when the receptor or receptor complex contained one of these genes, yielding 12 signaling pathways and 115 ligand–receptor pairs. Expression of a multisubunit receptor complex was defined as the minimum expression among its required subunits, consistent with complex-aware ligand–receptor representations (54).

Because the mass-action communication-probability model and cell-label permutation test implemented by CellChat were not run, this analysis was designated a “CellChatDB-guided ligand–receptor coexpression analysis” rather than a standard CellChat communication analysis. For each sample, source cell type, target cell type, and ligand–receptor pair, a geometric-mean score was calculated from non-negative pseudobulk logCPM values:


ligand−receptor coexpression score=√[max(L, 0)×max(R, 0)].

Scores were standardized to z scores within each dataset, cell-direction, and interaction and then averaged across regions from the same patient. The directions evaluated comprised each of the five microenvironmental cell types to malignant cells and malignant cells to each of those five cell types. A pathway score was calculated as the mean score of the ligand–receptor pairs assigned to that pathway. Patient-level linear models estimated the iCCA–HCC difference within each dataset, followed by inverse-variance fixed-effect pooling. BH adjustment was conducted separately at the pathway, ligand–receptor, and overall-network levels. Expression-based ligand–receptor analyses have important inferential distinctions from experimentally demonstrated physical signaling and were interpreted accordingly (55).

### Statistical analysis and reproducibility

Unless otherwise specified, all tests were two-sided. Categorical variables are reported as the number positive, number evaluable, and percentage; effects are reported as ORs or linear-model coefficients with 95% CIs. A prespecified q ≤ 0.10 threshold was used for discovery-stage candidate selection, and P < 0.05 for replication of the single external hypothesis. Raw P values and BH-adjusted q values are reported for TCGA, single-cell, and ligand–receptor analyses. The primary cancer-type comparisons were unadjusted. Age and sex were available for all 52 institutional patients, but etiology, cirrhosis status, and stage were not structured; with only 21 HCC and 11 CCA cases evaluable for the primary RTK–RAS comparison, no *post hoc* multivariable model was fitted. Although some MSK and TCGA clinical fields were available, etiology and stage were not sufficiently harmonized or complete across both cancer types for a common adjustment model; missing covariates were not imputed. Analyses and figures were generated locally in R 4.6.1; downloads, file verification, and delivery auditing used Python 3.13.13. Scripts, seeds, logs, software versions, and plotting-source data were retained.

### Ethics approval

The study was approved by the Ethics Committee of Yichang Central People’s Hospital (approval no. 2025-407-01). This retrospective study used only deidentified data generated during prior clinical care and genomic testing, involved no additional specimen collection, patient intervention, or follow-up, and received a waiver of informed consent from the ethics committee. The study was conducted in accordance with the ethical principles for medical research involving human participants (56). Public-cohort data were obtained from deidentified open-access databases and analyzed in accordance with their respective data-use policies.

## Results

### RTK–RAS pathway alterations were enriched in CCA and remained consistent across sensitivity analyses

The institutional cohort comprised 52 patients: 28 with HCC and 24 in the CCA working cohort, yielding 79 unique patient–positive-gene records. These full enrollment counts describe cohort composition. Primary gene and pathway inference was restricted to the broad-panel population (23 HCC and 13 CCA patients), with a separate evaluable denominator for each feature according to actual assay coverage. Among the 24 CCA cases, six reports explicitly stated iCCA and hepatic sampling supported an intrahepatic origin in five additional cases; these 11 patients formed the case-definition sensitivity population (**Figures 1A, B**).

**Figure 1:**
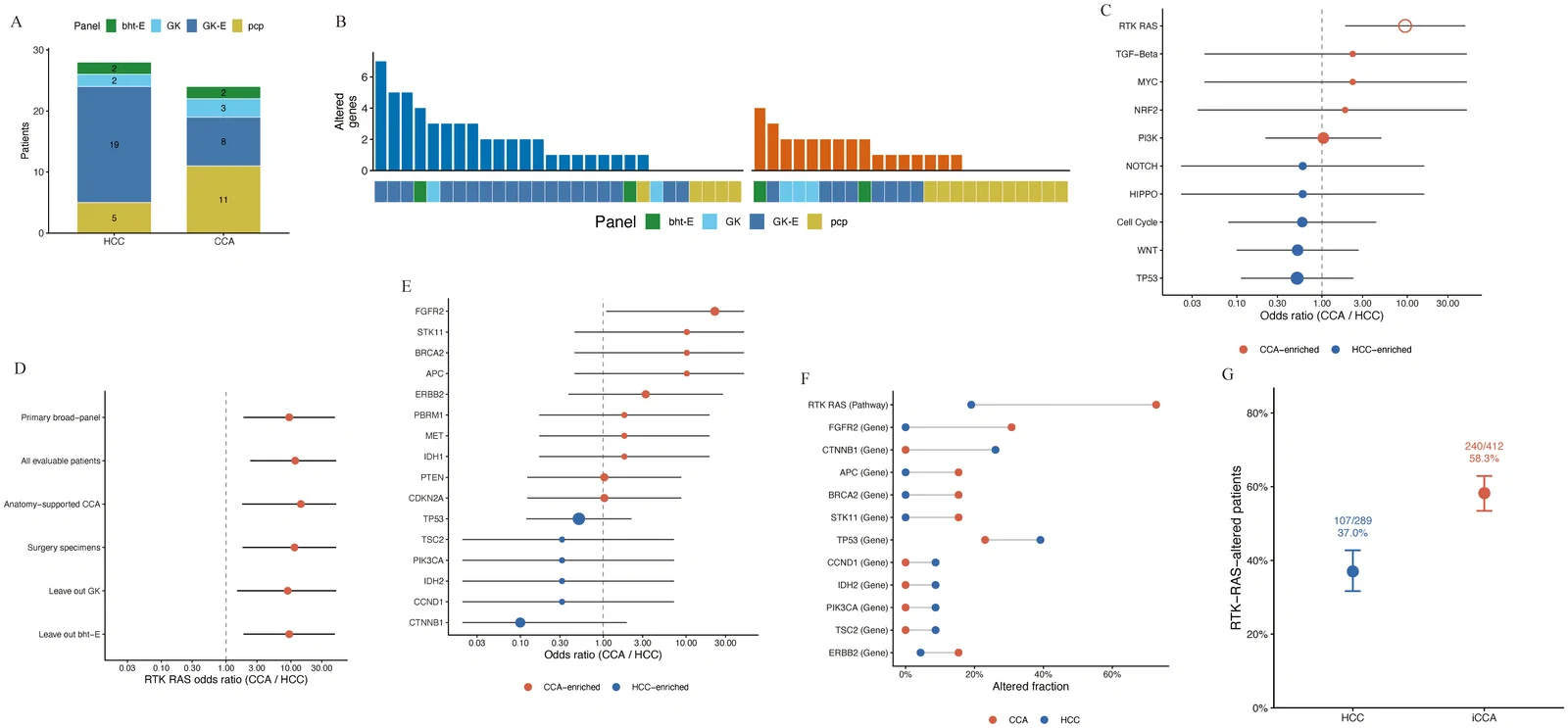
Unbiased pathway screening identifies RTK–RAS as the principal genomic difference between CCA and HCC. **(A)** Sequencing-panel composition of the full institutional cohort (28 HCC and 24 CCA patients). The stacked bars show GK, GK-E, bht-E, and pcp use. **(B)** Patient-level number of report-positive genes and panel assignment in all 52 patients. Each bar represents one patient; the cohort yielded 79 unique patient–positive-gene records. **(C)** Competition among the 10 fixed Sanchez-Vega oncogenic pathways in the broad-panel discovery population. The x-axis shows the Haldane–Anscombe-corrected OR for CCA versus HCC on a logarithmic scale; the dashed line denotes OR = 1, point size denotes total positive events, red indicates CCA enrichment, and blue indicates HCC enrichment. RTK–RAS alterations occurred in 4/21 HCC and 8/11 CCA cases (OR = 9.44; 95% CI, 1.87–47.70; Fisher P = 0.00601; BH q = 0.0601). **(D)** Prespecified sensitivity analyses of the locked RTK–RAS candidate. Points and error bars show corrected ORs and 95% CIs for the primary broad-panel analysis, all evaluable patients, the report-supported intrahepatic CCA subset, surgical specimens, and sequential panel exclusions. The main OR was 9.44; corresponding ORs were 11.67 for all evaluable patients, 14.26 for the report-supported intrahepatic subset, and 11.40 for surgical specimens. **(E)** Gene-level competition in the broad-panel population among 16 genes meeting the evaluability and event-count rules. Graphical encoding follows panel **(C)** FGFR2 showed the largest CCA-favoring effect (0/23 HCC vs. 4/13 CCA; OR = 22.26; 95% CI, 1.09–455.02; P = 0.0121; gene-family BH q = 0.194). **(F)** Alteration frequencies for the 12 highest-ranked gene and pathway features. Blue points denote HCC, red points denote CCA, and gray lines connect cancer-type frequencies for the same feature. RTK–RAS frequencies were 19.0% and 72.7%; FGFR2 frequencies were 0% and 30.8% in their respective evaluable broad-panel denominators. **(G)** Integrated RTK–RAS alteration prevalence in the independent MSK cohorts using the 38-gene common-coverage core and combining non-synonymous mutations, deep CNAs, and SVs. HCC: 107/289 (37.0%); iCCA: 240/412 (58.3%). Points show proportions and error bars show Wilson 95% CIs.

Among the 10 fixed oncogenic pathways, RTK–RAS showed the largest cancer-type difference and was the only pathway to reach the multiple-testing-adjusted screening threshold. Alterations were detected in 4/21 evaluable HCC cases (19.0%) and 8/11 evaluable CCA cases (72.7%), corresponding to a Haldane–Anscombe-corrected OR of 9.44 (95% CI, 1.87–47.70; P = 0.00601; BH-adjusted q = 0.0601 across 10 pathways; **Figure 1C**). The other nine pathways each had q = 1.00.

All sensitivity analyses yielded effects in the same direction. With all panels included and denominators based on actual assay coverage, RTK–RAS alteration rates were 4/21 in HCC and 10/13 in CCA (OR = 11.67; 95% CI, 2.37–57.36; P = 0.00139). Restriction to CCA cases with report-based support for an intrahepatic origin yielded 4/21 and 5/6 (OR = 14.26; 95% CI, 1.78–114.31; P = 0.00795), and restriction to surgical specimens yielded 3/12 and 10/12 (OR = 11.40; 95% CI, 1.80–72.15; P = 0.0123; **Figure 1D**). Sequential exclusion of each broad panel also retained the CCA-favoring direction.

Among 16 genes meeting the prespecified evaluability and event-count requirements, FGFR2 had the largest CCA-favoring effect: 0/23 evaluable HCC cases and 4/13 evaluable CCA cases (30.8%; OR = 22.26; 95% CI, 1.09–455.02; P = 0.0121; gene-family BH-adjusted q = 0.194; **Figure 1E**). These are broad-panel evaluable denominators; the full enrollment counts of 28 HCC and 24 CCA patients are not used as gene-level denominators when a gene was not covered. CTNNB1 ranked among the leading HCC-favoring events (**Figure 1F**). Together, the fixed pathway comparison and sensitivity analyses selected RTK–RAS for external validation.

### Enrichment of RTK–RAS pathway alterations in iCCA was replicated in an independent cohort

The external analysis included 412 primary iCCA tumors and 289 primary HCC tumors, with the comparison restricted to 38 RTK–RAS genes jointly covered by all four MSK-IMPACT panels. At least one core RTK–RAS event was detected in 240/412 iCCA cases (58.3%) and 107/289 HCC cases (37.0%), yielding an OR of 2.37 for iCCA relative to HCC (95% CI, 1.74–3.22; P = 3.12 × 10^−8^; **Figures 1G**, **2A**). The direction was concordant with the institutional cohort, replicating the RTK–RAS cancer-type difference in an independent genomic cohort.

**Figure 2:**
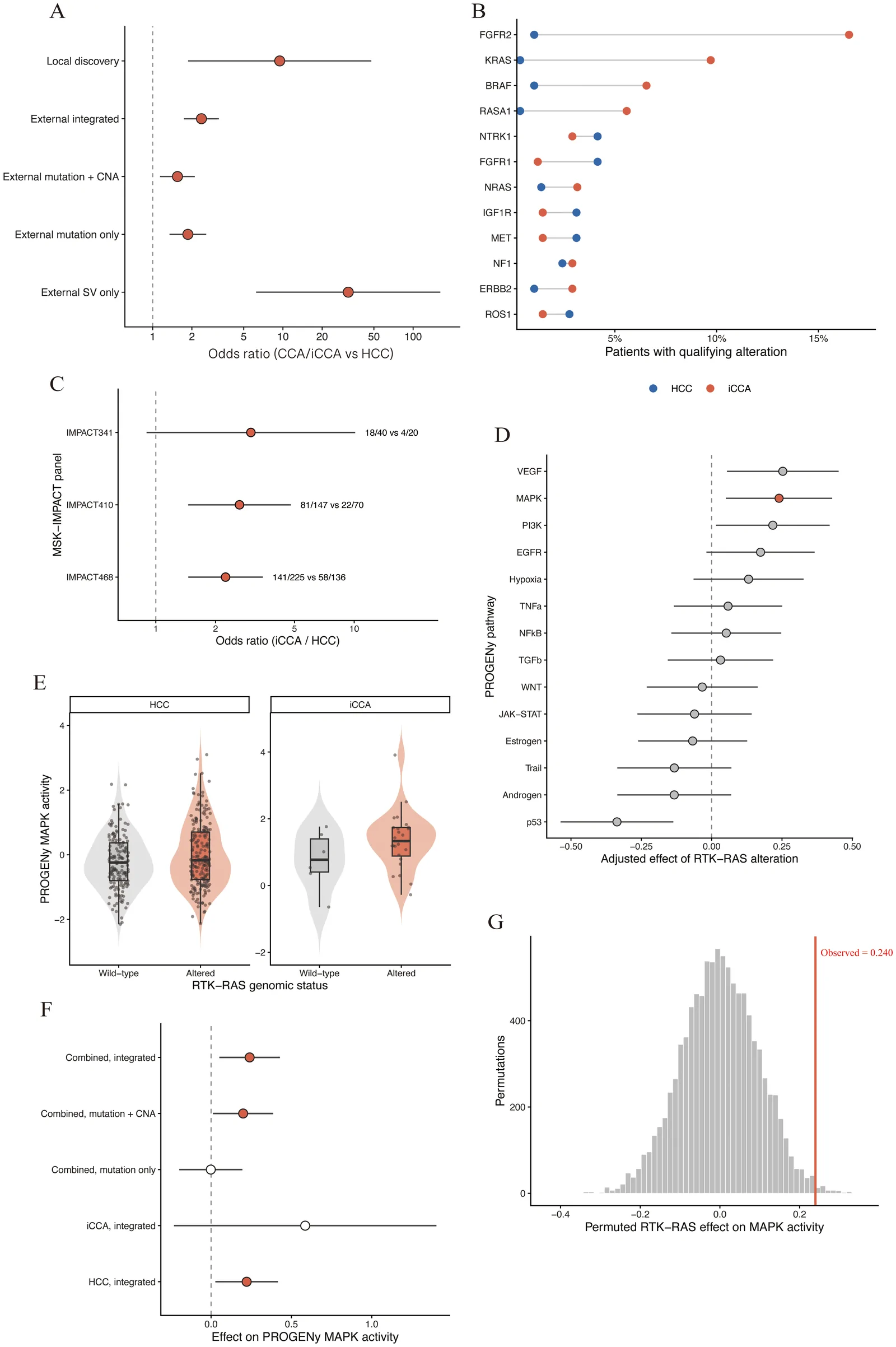
RTK–RAS alterations are replicated in the MSK cohort and associated with MAPK transcriptional activity in TCGA. **(A)** Forest plot comparing the institutional discovery effect with MSK external estimates under integrated and event-class-specific definitions. The x-axis is the OR for CCA/iCCA versus HCC on a logarithmic scale. The MSK integrated OR was 2.37 (95% CI, 1.74–3.22; P = 3.12 × 10^−8^); mutation-plus-CNA OR, 1.55 (P = 0.00530); mutation-only OR, 1.86 (P = 1.61 × 10^−4^); and SV-only OR, 31.74 (P = 2.72 × 10^−13^). **(B)** Patient-level frequencies of the RTK–RAS genes contributing most strongly to the MSK difference. Blue denotes HCC and red denotes iCCA; qualified events include non-synonymous mutations, deep CNAs, and SVs. FGFR2, KRAS, BRAF, RASA1, and other members contributed to the iCCA-side difference. **(C)** Panel-version-stratified MSK estimates. IMPACT341 included 18/40 iCCA versus 4/20 HCC cases (OR = 3.01); IMPACT410, 81/147 versus 22/70 (OR = 2.64); and IMPACT468, 141/225 versus 58/136 (OR = 2.25). The Cochran–Mantel–Haenszel common OR was 2.46 (95% CI, 1.75–3.44; P = 1.51 × 10^−7^). **(D)** RTK–RAS-status effects across all 14 PROGENy pathways in 393 TCGA tumors after adjustment for cancer type. Points and error bars show coefficients and 95% CIs; the dashed line denotes zero. The prespecified MAPK effect was 0.240 (95% CI, 0.051–0.428; P = 0.0129; BH q = 0.0600). **(E)** Patient-level PROGENy MAPK activity by integrated RTK–RAS status, displayed separately for HCC and iCCA. Violins show density, boxes show the median and interquartile range, and points represent patients. The cohort contained 229 altered and 164 unaltered tumors. **(F)** Sensitivity analyses for MAPK activity. Effects are shown for the pooled integrated model, mutation-plus-CNA model, mutation-only model, and cancer-type-specific integrated models. The iCCA-specific effect was 0.585 (95% CI, −0.231 to 1.402; P = 0.153), indicating a positive but imprecise within-iCCA estimate. **(G)** Null distribution from 10,000 within-cancer-type permutations of RTK–RAS status. The red line denotes the observed effect of 0.240; the two-sided empirical P value was 0.0113. Cancer-type-stratified bootstrap resampling (2,000 replicates) yielded a 95% CI of 0.052–0.421.

Decomposition by event class showed contributions from multiple alteration types. Non-synonymous mutations occurred in 170/412 iCCA cases (41.3%) and 79/289 HCC cases (27.3%; OR = 1.86; 95% CI, 1.35–2.57; P = 1.61 × 10^−4^). For mutations plus deep copy-number alterations, the OR was 1.55 (P = 0.00530). Structural variants occurred in 58/412 iCCA cases (14.1%) and 1/289 HCC case (0.35%; OR = 31.74; 95% CI, 6.23–161.69; P = 2.72 × 10^−13^), whereas copy-number alterations alone did not differ significantly (OR = 0.69; P = 0.145; **Figure 2A**). FGFR2-related structural variants were a major iCCA-side component, alongside KRAS, BRAF, RASA1, and other pathway events (**Figure 2B**). Among 50 iCCA patients with an FGFR2-involving structural-variant record, seven had an explicitly Archer-labeled fusion annotation; the public files did not document uniform orthogonal confirmation for the remaining records.

Replication remained consistent across panel strata. The ORs within the IMPACT341, IMPACT410, and IMPACT468 strata were 3.01, 2.64, and 2.25, respectively, each favoring a higher prevalence in iCCA. The Cochran–Mantel–Haenszel common OR stratified by panel version was 2.46 (95% CI, 1.75–3.44; P = 1.51 × 10^−7^; **Figure 2C**). The direction and magnitude of the effect remained consistent before and after stratification by panel version.

### RTK–RAS alterations were associated with increased MAPK transcriptional activity

The matched genomic–transcriptomic TCGA analysis included 393 patients, comprising 363 with HCC and 30 with iCCA; 229 patients harbored at least one integrated RTK–RAS event, whereas no such event was detected in 164 patients. After adjustment for cancer type, PROGENy MAPK activity was 0.240 standardized units higher in the altered group than in the unaltered group (95% CI, 0.051–0.428; P = 0.0129; **Figures 2D, E**). Among the 14 PROGENy pathways evaluated in the same model, the adjusted q value for MAPK was 0.0600.

When the event definition was restricted to mutations plus deep copy-number alterations, the MAPK effect remained positive (0.199; 95% CI, 0.012–0.386; P = 0.0370); with non-synonymous mutations alone, it was near zero (−0.002; P = 0.984). Cancer-type-specific effects were 0.221 in HCC (95% CI, 0.027–0.416; P = 0.0261) and 0.585 in iCCA (95% CI, −0.231 to 1.402; P = 0.153; **Figure 2F**). Thus, the primary evidence for association came from the pooled model adjusted for cancer type; the within-iCCA estimate was positive but imprecise and did not independently reach statistical significance. Ten thousand within-cancer-type permutations yielded an empirical P = 0.0113, and 2,000 cancer-type-stratified bootstrap resamples yielded a 95% CI of 0.052–0.421 (**Figure 2G**). The attenuation in the mutation-only model indicates that the observed cohort-level association depended on the integrated event definition.

### Increased MAPK activity in iCCA was localized predominantly to malignant cells

GSE151530 and GSE189903 were used to localize the cellular source of the MAPK difference between cancer types. GSE151530 included 17,164 malignant cells (15,723 from HCC and 1,441 from iCCA), whereas GSE189903 included 1,574 malignant cells (1,134 from HCC and 440 from iCCA; **Figures 3A, B**).

**Figure 3:**
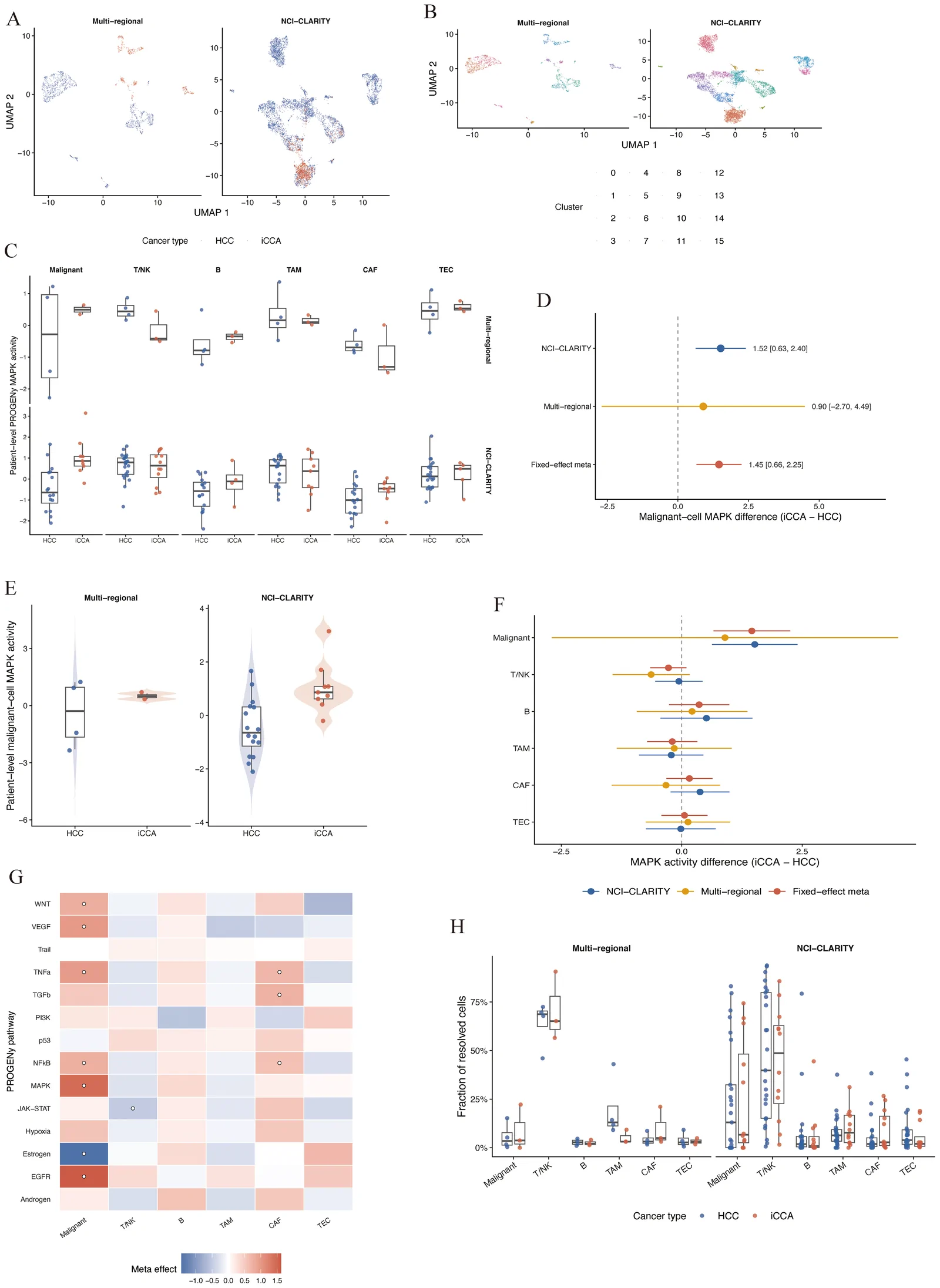
Increased MAPK activity in iCCA is localized predominantly to malignant cells at single-cell resolution. **(A)** Separate malignant-cell UMAPs for GSE151530 and GSE189903, colored by cancer type. GSE151530 contained 17,164 malignant cells (15,723 HCC and 1,441 iCCA); GSE189903 contained 1,574 (1,134 HCC and 440 iCCA). Coordinates were not integrated across datasets, and cells were not used as independent statistical units. **(B)** The same malignant-cell UMAP coordinates colored by within-dataset transcriptional clusters. Cluster labels describe within-dataset states and are not aligned across cohorts. **(C)** Patient-level pseudobulk PROGENy MAPK scores across malignant cells, T/NK cells, B cells, TAMs, CAFs, and TECs. Each point represents one patient; multiregional observations were averaged within patient before inference. **(D)** Focused malignant-cell forest plot. GSE151530: effect = 1.516 (95% CI, 0.630–2.402; HCC n = 16, iCCA n = 9); GSE189903: effect = 0.897 (95% CI, −2.697 to 4.492; HCC n = 4, iCCA n = 2). The fixed-effect estimate was 1.455 (95% CI, 0.658–2.252; P = 3.46 × 10^−4^; q = 0.00208; I^2^ = 0%). Inverse-variance weights were 90.1% and 9.9%, respectively. **(E)** Patient-level malignant-cell MAPK score distributions by dataset and cancer type. Violins and boxes display the distribution, median, and interquartile range; each point represents one patient after within-patient aggregation. **(F)** Dataset-specific and inverse-variance pooled iCCA-minus-HCC MAPK effects across six cell types. Blue denotes GSE151530, orange denotes GSE189903, and red denotes the pooled estimate; error bars are 95% CIs. Only the malignant-cell pooled effect met the six-cell-type adjusted threshold. **(G)** Cross-dataset fixed effects for all 14 PROGENy pathways across six cell types. Red indicates higher activity in iCCA, blue indicates higher activity in HCC, and outlined symbols denote combinations meeting the global multiple-testing threshold. Malignant-cell MAPK shows a significant positive effect in the full pathway-by-cell-type comparison. **(H)** Patient-level proportions of the six analyzed cell compartments in each dataset. GSE151530 included 25 HCC and 12 iCCA patients for composition analysis; GSE189903 included four HCC and three iCCA patients. No composition comparison reached the adjusted threshold (all q = 0.877 in GSE151530; all q ≥ 0.809 in GSE189903).

In GSE151530, the malignant-cell comparison retained 16 HCC and nine iCCA patients; MAPK activity was 1.516 standardized units higher in iCCA (95% CI, 0.630–2.402; P = 0.00175; BH-adjusted q = 0.0105 across six cell types). In GSE189903, four HCC and two iCCA patients met the malignant-cell criterion after quality control. Its effect was also positive (0.897) but imprecise (95% CI, −2.697 to 4.492; P = 0.526; **Figures 3C–E**).

The fixed-effect pooled estimate was 1.455 (95% CI, 0.658–2.252; P = 3.46 × 10^−4^; BH-adjusted q = 0.00208 across six cell types; global q = 0.00582 across 84 pathway-by-cell-type comparisons; I^2^ = 0%; **Figures 3F, G**). The normalized inverse-variance weights were 90.1% for GSE151530 and 9.9% for GSE189903, so the smaller cohort provided directional support rather than a precise standalone replication. MAPK effects in T/NK cells, B cells, TAMs, CAFs, and TECs did not reach q < 0.05 after correction across six cell types. Cell-type composition also showed no adjusted difference (all q = 0.877 in GSE151530 and q ≥ 0.809 in GSE189903; **Figure 3H**). These findings support predominant localization of the cancer-type MAPK difference to the malignant-cell compartment. RTK–RAS receptor, core kinase, and negative-regulator expression across the six cell types is shown in **Figure 4A**.

**Figure 4:**
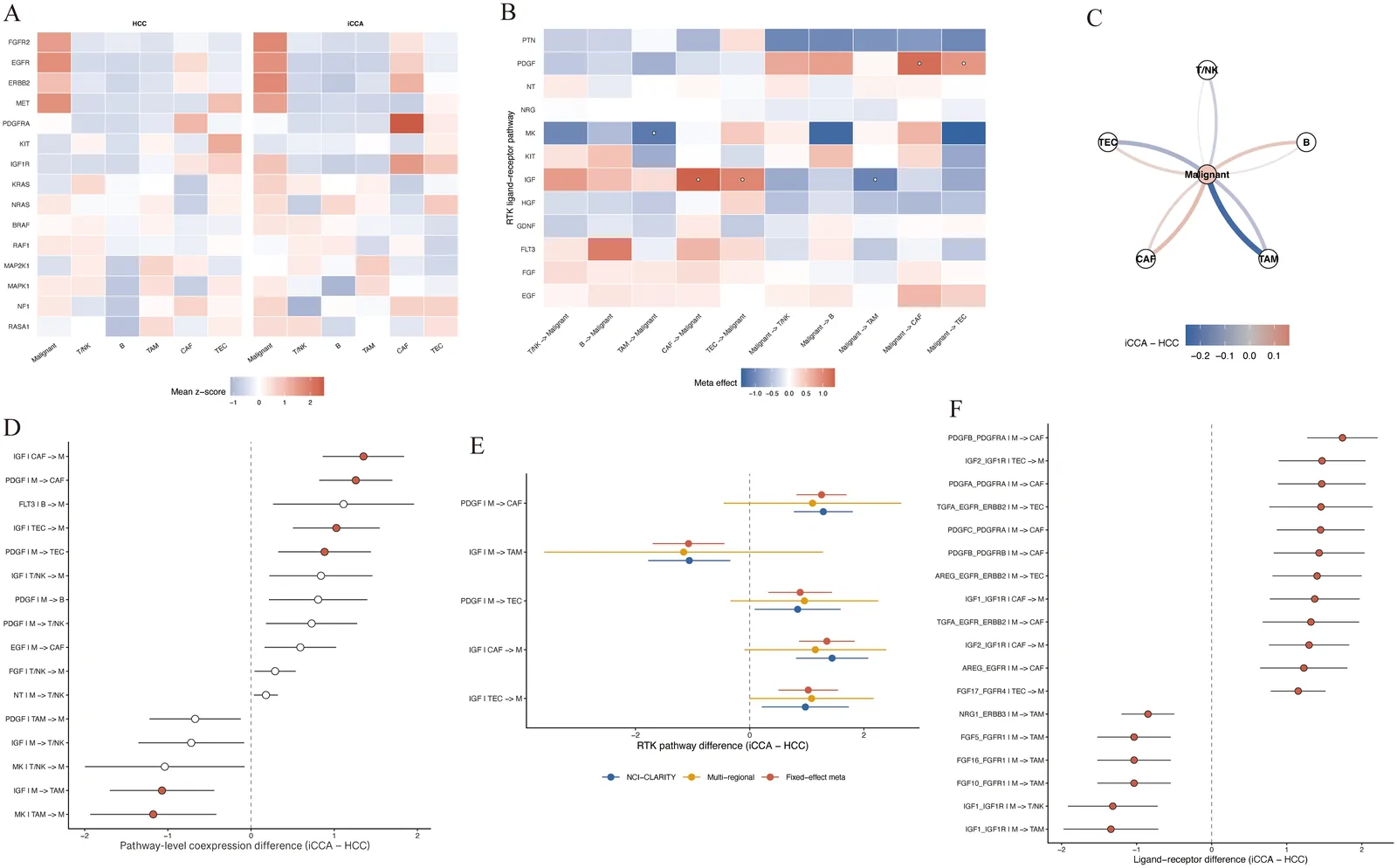
Cell-type-resolved analysis identifies the RTK-related tumor–stromal signaling context of the MAPK phenotype. **(A)** Mean patient-level expression z scores for 15 prespecified RTK–RAS genes across malignant cells, T/NK cells, B cells, TAMs, CAFs, and TECs, shown separately for HCC and iCCA. The genes include receptors, core kinases, and negative regulators and provide the expression basis for ligand–receptor evaluation. **(B)** Overview of iCCA-minus-HCC effects for 12 CellChatDB secreted-signaling pathways across the 10 bidirectional routes between malignant cells and five microenvironmental compartments. Red indicates higher coexpression in iCCA, blue indicates higher coexpression in HCC, and outlined symbols denote BH q < 0.10. **(C)** Overall RTK-related network after aggregating 115 fixed ligand–receptor scores within patient and direction. Each paired curve represents the two separately estimated source-to-target routes between malignant cells and one microenvironmental compartment; line width denotes the absolute pooled effect, color denotes the iCCA-minus-HCC effect, and transparency reflects statistical evidence. Detailed route directions are shown in panel **(B)**. This is a transcript-level coexpression summary, not a physical communication model. **(D)** Forest plot of the 16 highest-ranked RTK-related pathway-by-direction effects. Malignant-cell-to-CAF PDGF had an effect of 1.259 (95% CI, 0.820–1.698; P = 1.86 × 10^−8^; q = 2.23 × 10^−6^); CAF-to-malignant-cell IGF, 1.352 (95% CI, 0.864–1.840; P = 5.57 × 10^−8^; q = 3.34 × 10^−6^); and TEC-to-malignant-cell IGF, 1.026 (q = 0.00462). M denotes malignant cells. **(E)** Dataset-specific and pooled estimates for all pathway-by-direction combinations meeting pathway-level BH q < 0.05. The figure displays every qualifying combination without manual selection and distinguishes concordant directional support from the pooled estimate. **(F)** Forest plot of the 18 highest-ranked prespecified ligand–receptor-by-direction effects. Malignant-cell-to-CAF PDGFB–PDGFRA showed the largest pooled effect (1.743; 95% CI, 1.273–2.212; P = 3.37 × 10^−13^; q = 3.67 × 10^−10^), followed by IGF2–IGF1R and other PDGFRA-family combinations.

### PDGF and IGF pathway-level coexpression was increased between malignant and stromal cells

The CellChatDB prior yielded 12 secreted-signaling pathways and 115 ligand–receptor combinations; the cellular directions and overall network of RTK-related signaling are shown in **Figures 4B, C**. At the pathway level, platelet-derived growth factor (PDGF) coexpression from malignant cells to CAFs was increased in iCCA, with a pooled cross-dataset effect of 1.259 (95% CI, 0.820–1.698; P = 1.86 × 10^−8^; BH-adjusted q across 120 pathway-by-direction comparisons = 2.23 × 10^−6^; **Figures 4D, E**). In the opposite direction, the effect for insulin-like growth factor (IGF) coexpression from CAFs to malignant cells was 1.352 (95% CI, 0.864–1.840; P = 5.57 × 10^−8^; q = 3.34 × 10^−6^), and the effect from TECs to malignant cells was 1.026 (95% CI, 0.504–1.547; q = 0.00462).

### PDGF–PDGFRA and IGF–IGF1R coexpression axes were consistently increased across single-cell datasets

Ligand–receptor decomposition localized the PDGF pattern to the PDGFRA receptor family (**Figure 4F**). For malignant-cell-to-CAF PDGFB–PDGFRA coexpression, the pooled effect was 1.743 (95% CI, 1.273–2.212; P = 3.37 × 10^−13^; BH-adjusted q = 3.67 × 10^−10^ across 1,090 ligand–receptor comparisons). The effect was 1.796 in GSE151530 (95% CI, 1.267–2.324; P = 1.57 × 10^−6^) and 1.181 in GSE189903 (95% CI, −1.076 to 3.437; P = 0.220; **Figures 5A, B**). Pooled effects for PDGFA–PDGFRA and PDGFC–PDGFRA were 1.466 and 1.451, with q = 2.44 × 10^−4^ and 2.45 × 10^−4^, respectively (**Figure 5C**).

**Figure 5:**
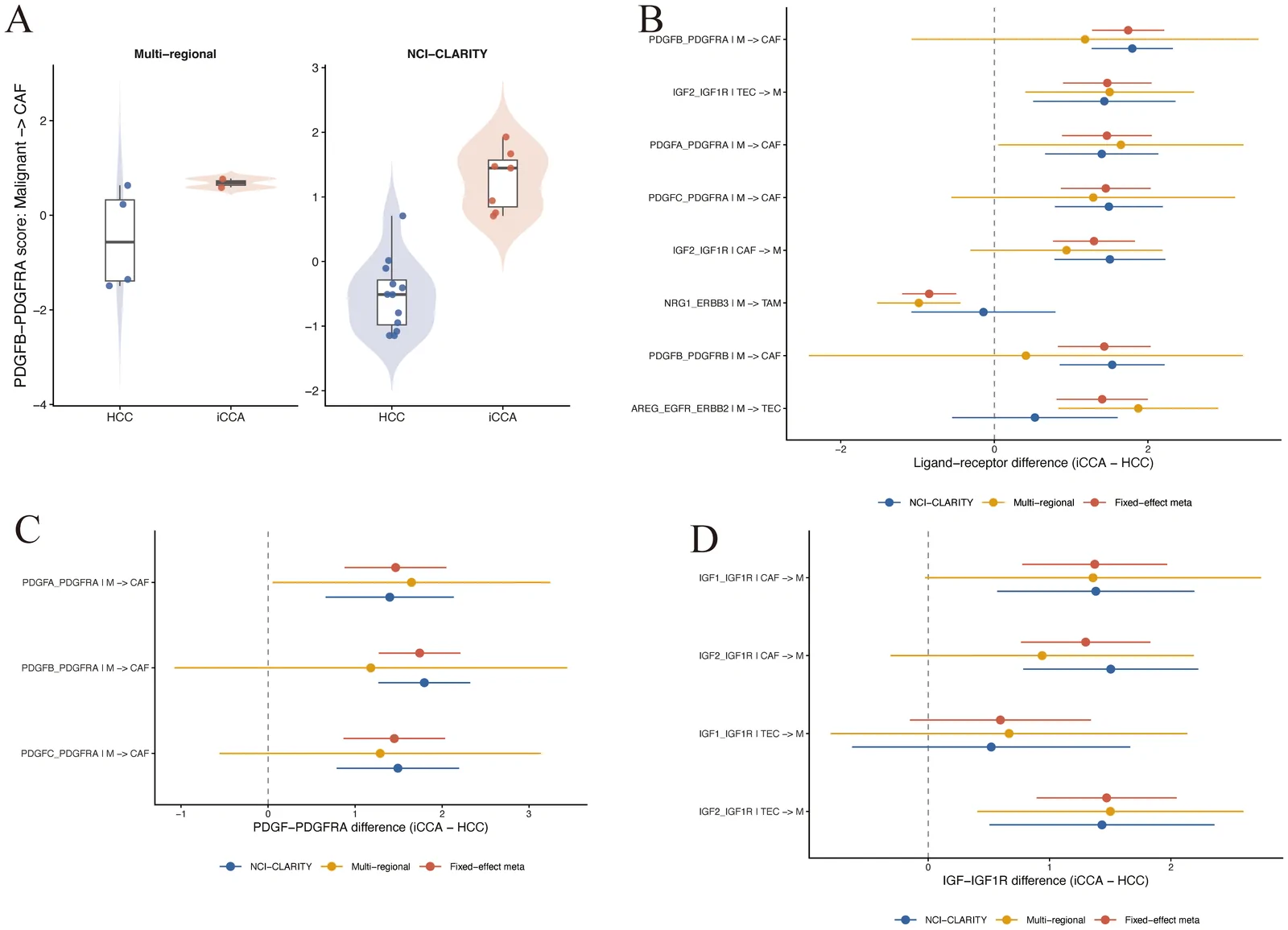
PDGF–PDGFRA and IGF–IGF1R coexpression axes are consistently increased across single-cell datasets. **(A)** Patient-level malignant-cell-to-CAF PDGFB–PDGFRA coexpression scores by dataset and cancer type. GSE151530 included 12 HCC and seven iCCA patients (effect = 1.796; 95% CI, 1.267–2.324; P = 1.57 × 10^−6^); GSE189903 included four HCC and two iCCA patients (effect = 1.181). The pooled effect was 1.743 (95% CI, 1.273–2.212; q = 3.67 × 10^−10^). **(B)** Dataset-specific and pooled effects for the eight highest-ranked ligand–receptor combinations that were estimable in both datasets and met ligand–receptor-level BH q < 0.001. Blue denotes GSE151530, orange denotes GSE189903, and red denotes the pooled fixed effect. **(C)** Malignant-cell-to-CAF PDGFRA-family coexpression. Pooled effects were 1.466 for PDGFA–PDGFRA (95% CI, 0.881–2.051; q = 2.44 × 10^−4^), 1.743 for PDGFB–PDGFRA (95% CI, 1.273–2.212; q = 3.67 × 10^−10^), and 1.451 for PDGFC–PDGFRA (95% CI, 0.867–2.035; q = 2.45 × 10^−4^). **(D)** CAF/TEC-to-malignant-cell IGF1R coexpression. Pooled effects were 1.373 for CAF-derived IGF1–IGF1R (95% CI, 0.776–1.970; q = 7.23 × 10^−4^), 1.298 for CAF-derived IGF2–IGF1R (95% CI, 0.765–1.831; q = 3.36 × 10^−4^), and 1.471 for TEC-derived IGF2–IGF1R (95% CI, 0.893–2.048; q = 2.15 × 10^−4^).

IGF coexpression directed toward malignant cells was concentrated on IGF1R. The pooled effect for TEC-derived IGF2–IGF1R was 1.471 (95% CI, 0.893–2.048; q = 2.15 × 10^−4^). Pooled effects for CAF-derived IGF1–IGF1R and IGF2–IGF1R were 1.373 and 1.298, with q = 7.23 × 10^−4^ and 3.36 × 10^−4^, respectively (**Figure 5D**). Together, the data describe a malignant-cell-to-CAF PDGF–PDGFRA coexpression pattern and CAF/TEC-to-malignant-cell IGF–IGF1R coexpression patterns; they do not establish physical ligand–receptor binding or universal communication networks.

## Discussion

Primary liver cancers are not a homogeneous group defined solely by their anatomic origin in the liver. HCC and iCCA exhibit molecular features rooted in hepatocytic and cholangiocytic lineages, respectively: HCC is commonly characterized by TP53, WNT/β-catenin, and cell-cycle alterations, whereas iCCA is enriched for FGFR2 rearrangements, IDH1/2 mutations, and growth-signaling events involving KRAS and BRAF (2, 4, 5, 7, 8, 12, 13). Previous studies have established the driver-gene landscapes and molecular subclasses of the two cancers, but most have focused on heterogeneity within a single tumor type (14, 29). An integrative question therefore remains: whether HCC and iCCA differ at the level of a consistently defined oncogenic pathway across testing platforms and whether such a genomic difference corresponds to a coherent transcriptional output and a defined cellular source.

The principal contribution of this study is not the rediscovery of FGFR2, KRAS, BRAF, or other established single-gene differences. It is the connection of four analytically distinct levels: a panel-aware pathway difference selected from 10 fixed oncogenic pathways; replication of that difference in an independent genomic cohort; association of integrated RTK–RAS status with MAPK transcriptional output; and localization of the intertumoral MAPK difference predominantly to malignant cells. The findings therefore recast a heterogeneous list of mutations, copy-number alterations, and structural variants as multiple genomic inputs associated with a shared downstream transcriptional phenotype, while retaining alteration-specific interpretation for clinical decisions.

Pathway-level comparison was central to this interpretation. Pan-cancer studies show that receptor fusions, activating mutations, copy-number alterations, and loss of negative regulators can perturb the same oncogenic pathway, so single-gene frequencies alone do not capture pathway convergence (27, 28). FGFR2 had the largest CCA-favoring gene-level effect in the institutional cohort, but the external RTK–RAS difference also involved KRAS, BRAF, RASA1, and other members. This pattern accords with the proliferation class of iCCA described by Sia et al. and with the selective distribution of FGFR2, KRAS/BRAF, and epigenetic drivers across biliary tract cancer subgroups (12–14). RTK–RAS enrichment is therefore a population-level feature generated by heterogeneous events rather than a uniform molecular subtype defined by one alteration.

Event-class decomposition further explains the relationship between genomic status and transcriptional output. Structural variants contributed strongly to RTK–RAS enrichment in the MSK iCCA cohort. In TCGA, the integrated status incorporating mutations, deep copy-number alterations, and structural variants was associated with MAPK activity, whereas the mutation-only association was absent. Experimental studies show that FGFR2 fusions can activate MAPK, while proteogenomic data demonstrate driver-specific downstream states in iCCA (16, 29). Pathway convergence therefore does not imply biological equivalence among all component events; it indicates that different entry points can remodel a shared growth-signaling network. Because orthogonal fusion confirmation was not uniformly documented in the public MSK records, the structural-variant component should be interpreted as panel-detected rather than uniformly RNA- or FISH-confirmed (43).

The anatomic classification of the institutional cohort requires separate interpretation. Its biliary cases are reported as a CCA working cohort because only 11/24 had report-based evidence supporting an intrahepatic origin. iCCA and extrahepatic CCA have materially different genomic landscapes, particularly for FGFR2 alterations (13, 20, 21). The institutional sensitivity analysis restricted to report-supported intrahepatic cases retained the same direction, but it does not by itself provide a precise iCCA-specific estimate. The inference specific to iCCA is supported chiefly by the independently classified MSK and TCGA cohorts; the institutional result should not be extrapolated to all anatomic CCA subtypes.

Patient-level single-cell analyses helped localize the MAPK phenotype. GSE151530 produced the precise malignant-cell estimate, whereas GSE189903 contributed 9.9% of the inverse-variance weight and served as directional support. The use of patient-level pseudobulk and within-patient aggregation of multiple regions prevented cell number and regional sampling from inflating the biological replication. The suggested GSE125449 dataset could not provide an independent third estimate because all 19 patient identifiers overlapped with GSE151530 (45). The pooled result therefore supports predominant malignant-cell localization across the two analyzed datasets, but its precision is governed mainly by GSE151530 and does not establish that every iCCA subtype has elevated MAPK activity.

The stroma-rich histologic architecture of iCCA provides a biological context for the observed PDGF and IGF coexpression patterns. Prior single-cell and experimental studies have identified heterogeneous CAF populations and growth-factor programs in iCCA (32, 33). In the present data, malignant-cell-to-CAF PDGF–PDGFRA and CAF/TEC-to-malignant-cell IGF–IGF1R were reproducible expression pairings across the two analyzed datasets. These results are best viewed as focused hypotheses about the stromal context accompanying the malignant-cell MAPK phenotype, rather than as proof of universal or physically active communication circuits.

Within the PDGF axis, the iCCA-associated increase was directed predominantly from malignant cells to CAFs, with PDGFB–PDGFRA showing the largest effect and PDGFA–PDGFRA and PDGFC–PDGFRA changing in the same direction. Cadamuro et al. showed that tumor-derived PDGF-D can recruit fibroblasts through CAF PDGFRβ and promote CCA-associated lymphangiogenesis (57, 58). The common element is the tumor-to-fibroblast direction of PDGF-family signaling; the receptor branch differs, because our coexpression pattern centered on PDGFRA rather than the functionally tested PDGFRβ axis. This distinction makes PDGFA/B/C–PDGFRA a testable extension of prior biology, not a demonstrated mechanism.

The IGF direction complemented the PDGF pattern: increased coexpression arose from CAFs or TECs and pointed toward IGF1R in malignant cells. Vaquero et al. showed that stromal IGF2 can augment insulin receptor (IR)/IGF1R signaling in CCA cells and contribute to adaptation during EGFR inhibition (59). This functional evidence is directionally consistent with the observed CAF/TEC-derived IGF1/2–malignant-cell IGF1R coexpression pattern. Spatial colocalization, protein activation, and perturbation studies are nevertheless required before these transcript-level pairings can be interpreted as operative intercellular signaling.

The clinical implication concerns how genomic reports are summarized, not the proposal of a new companion diagnostic. FGFR2 fusion/rearrangement-positive iCCA and BRAF V600E-mutated biliary tract cancers already have established targeted-treatment evidence (24–26). The present pathway framework complements those alteration-specific decisions by showing that mutations, copy-number alterations, and structural variants distributed across multiple RTK–RAS genes can be evaluated together when describing population-level molecular differences. In real-world datasets assembled from non-identical panels, explicit coverage-aware denominators also prevent untested genes from being misclassified as wild type. Pathway burden should therefore be used for systems-level cohort interpretation, while treatment selection remains anchored to the exact gene, variant, alteration class, and approved clinical evidence.

Taken together, the data support a higher burden of heterogeneous RTK–RAS alterations in CCA/iCCA than in HCC and an association between integrated RTK–RAS status and MAPK transcriptional output. Patient-level single-cell analysis places the cancer-type MAPK difference predominantly in malignant cells, while PDGF–PDGFRA and IGF–IGF1R provide candidate stromal coexpression contexts. This evidence chain is consistent with pathway convergence across alteration classes, but it does not replace gene-specific therapeutic interpretation or establish causal cell–cell signaling.

## Conclusion

Integration of institutional tumor tissue genomic testing with independent genomic, bulk-transcriptomic, and single-cell datasets supports a higher burden of RTK–RAS pathway alterations in CCA/iCCA than in HCC. The association of integrated RTK–RAS status with MAPK activity and the predominant localization of the intertumoral MAPK difference to malignant cells provide a pathway-level link between heterogeneous genomic events and transcriptional phenotype. PDGF–PDGFRA and IGF–IGF1R coexpression patterns offer focused hypotheses for future spatial and functional validation.

## Limitations and future directions

The principal limitations are the small single-center discovery cohort, non-identical clinical panels, and incomplete anatomic classification of the institutional CCA cases. Age and sex were available, but etiology, cirrhosis status, and stage were unavailable or insufficiently harmonized across cohorts, precluding a common adjusted analysis. The primary institutional comparison therefore remains a panel-aware unadjusted contrast, and the iCCA-specific inference depends chiefly on the externally classified MSK and TCGA cohorts. Public MSK annotations also did not document uniform orthogonal confirmation of structural variants. The TCGA iCCA group was small, and its within-cancer estimate was imprecise. Single-cell precision was dominated by GSE151530; GSE189903 retained only two iCCA patients for the malignant-cell comparison and contributed 9.9% of the pooled weight. GSE125449 could not be added as an independent cohort because its patients overlapped with GSE151530. The ligand–receptor analysis measured transcript-level coexpression rather than physical communication and requires validation by larger independent cohorts, spatial assays, protein-level measurements, and functional perturbation. Treatment response, recurrence, and survival data were unavailable, so predictive and prognostic implications were not assessed.

## Data Availability

The analysis scripts, prespecified analysis documents, aggregate institutional results, public-cohort intermediate tables, processed single-cell pseudobulk objects, plotting data, and software-session records supporting the conclusions of this article are publicly available in Zenodo at https://doi.org/10.5281/zenodo.22679838. Public datasets analyzed in this study are available from cBioPortal (ihch_msk_2021, hcc_msk_2024, TCGA-LIHC, and TCGA-CHOL) and the Gene Expression Omnibus (GSE151530 and GSE189903). Individual-level institutional data and source clinical reports are not publicly available because they contain potentially identifiable clinical information and the ethics approval does not authorize unrestricted public sharing. Requests for controlled access may be directed to the corresponding author and are subject to ethics, institutional, and data-use approval.

## References

[1] Sung H Ferlay J Siegel RL Laversanne M Soerjomataram I Jemal A . Global cancer statistics 2020: GLOBOCAN estimates of incidence and mortality worldwide for 36 cancers in 185 countries. CA: A Cancer J For Clin. (2021) 71:209–49. doi: 10.3322/caac.21660 33538338

[2] Llovet JM Kelley RK Villanueva A Singal AG Pikarsky E Roayaie S . Hepatocellular carcinoma. Nat Rev Dis Primers. (2021) 7:6. doi: 10.1038/s41572-020-00240-3 33479224 PMC13537433

[3] Forner A Reig M Bruix J . Hepatocellular carcinoma. Lancet (London England). (2018) 391:1301–14. doi: 10.1016/S0140-6736(18)30010-2 29307467

[4] Banales JM Marin JJG Lamarca A Rodrigues PM Khan SA Roberts LR . Cholangiocarcinoma 2020: The next horizon in mechanisms and management. Nat Rev Gastroenterol Hepatol. (2020) 17:557–88. doi: 10.1038/s41575-020-0310-z 32606456 PMC7447603

[5] Brindley PJ Bachini M Ilyas SI Khan SA Loukas A Sirica AE . Cholangiocarcinoma. Nat Rev Dis Primers. (2021) 7:65. doi: 10.1038/s41572-021-00300-2 34504109 PMC9246479

[6] Hoadley KA Yau C Hinoue T Wolf DM Lazar AJ Drill E . Cell-of-origin patterns dominate the molecular classification of 10,000 tumors from 33 types of cancer. Cell. (2018) 173:291–304.e6. doi: 10.1016/j.cell.2018.03.022 29625048 PMC5957518

[7] Cancer Genome Atlas Research Network . Comprehensive and integrative genomic characterization of hepatocellular carcinoma. Cell. (2017) 169:1327–1341.e23. doi: 10.1016/j.cell.2017.05.046 28622513 PMC5680778

[8] Farshidfar F Zheng S Gingras MC Newton Y Shih J Robertson AG . Integrative genomic analysis of cholangiocarcinoma identifies distinct IDH-mutant molecular profiles. Cell Rep. (2017) 19:2878–80. doi: 10.1016/j.celrep.2017.06.008 28658632 PMC6141445

[9] Totoki Y Tatsuno K Covington KR Ueda H Creighton CJ Kato M . Trans-ancestry mutational landscape of hepatocellular carcinoma genomes. Nat Genet. (2014) 46:1267–73. doi: 10.1038/ng.3126 25362482

[10] Schulze K Imbeaud S Letouzé E Alexandrov LB Calderaro J Rebouissou S . Exome sequencing of hepatocellular carcinomas identifies new mutational signatures and potential therapeutic targets. Nat Genet. (2015) 47:505–11. doi: 10.1038/ng.3252 25822088 PMC4587544

[11] Fujimoto A Furuta M Totoki Y Tsunoda T Kato M Shiraishi Y . Whole-genome mutational landscape and characterization of noncoding and structural mutations in liver cancer. Nat Genet. (2016) 48:500–9. doi: 10.1038/ng.3547 27064257

[12] Nakamura H Arai Y Totoki Y Shirota T Elzawahry A Kato M . Genomic spectra of biliary tract cancer. Nat Genet. (2015) 47:1003–10. doi: 10.1038/ng.3375 26258846

[13] Jusakul A Cutcutache I Yong CH Lim JQ Huang MN Padmanabhan N . Whole-genome and epigenomic landscapes of etiologically distinct subtypes of cholangiocarcinoma. Cancer Discov. (2017) 7:1116–35. doi: 10.1158/2159-8290.CD-17-0368 28667006 PMC5628134

[14] Sia D Hoshida Y Villanueva A Roayaie S Ferrer J Tabak B . Integrative molecular analysis of intrahepatic cholangiocarcinoma reveals 2 classes that have different outcomes. Gastroenterology. (2013) 144:829–40. doi: 10.1053/j.gastro.2013.01.001 23295441 PMC3624083

[15] Sia D Losic B Moeini A Cabellos L Hao K Revill K . Massive parallel sequencing uncovers actionable FGFR2-PPHLN1 fusion and ARAF mutations in intrahepatic cholangiocarcinoma. Nat Commun. (2015) 6:6087. doi: 10.1038/ncomms7087 25608663

[16] Arai Y Totoki Y Hosoda F Shirota T Hama N Nakamura H . Fibroblast growth factor receptor 2 tyrosine kinase fusions define a unique molecular subtype of cholangiocarcinoma. Hepatol (Baltimore Md). (2014) 59:1427–34. doi: 10.1002/hep.26890 24122810

[17] Jiao Y Pawlik TM Anders RA Selaru FM Streppel MM Lucas DJ . Exome sequencing identifies frequent inactivating mutations in BAP1, ARID1A and PBRM1 in intrahepatic cholangiocarcinomas. Nat Genet. (2013) 45:1470–3. doi: 10.1038/ng.2813 24185509 PMC4013720

[18] Chan-On W Nairismägi ML Ong CK Lim WK Dima S Pairojkul C . Exome sequencing identifies distinct mutational patterns in liver fluke-related and non-infection-related bile duct cancers. Nat Genet. (2013) 45:1474–8. doi: 10.1038/ng.2806 24185513

[19] Ong CK Subimerb C Pairojkul C Wongkham S Cutcutache I Yu W . Exome sequencing of liver fluke-associated cholangiocarcinoma. Nat Genet. (2012) 44:690–3. doi: 10.1038/ng.2273 22561520

[20] Lowery MA Ptashkin R Jordan E Berger MF Zehir A Capanu M . Comprehensive molecular profiling of intrahepatic and extrahepatic cholangiocarcinomas: Potential targets for intervention. Clin Cancer Res: Off J Am Assoc For Cancer Res. (2018) 24:4154–61. doi: 10.1158/1078-0432.CCR-18-0078 29848569 PMC6642361

[21] Wardell CP Fujita M Yamada T Simbolo M Fassan M Karlic R . Genomic characterization of biliary tract cancers identifies driver genes and predisposing mutations. J Hepatol. (2018) 68:959–69. doi: 10.1016/j.jhep.2018.01.009 29360550

[22] Javle M Bekaii-Saab T Jain A Wang Y Kelley RK Wang K . Biliary cancer: Utility of next-generation sequencing for clinical management. Cancer. (2016) 122:3838–47. doi: 10.1002/cncr.30254 27622582

[23] European Association for the Study of the Liver . EASL-ILCA clinical practice guidelines on the management of intrahepatic cholangiocarcinoma. J Hepatol. (2023) 79:181–208. doi: 10.1016/j.jhep.2023.03.010 37084797

[24] Abou-Alfa GK Sahai V Hollebecque A Vaccaro G Melisi D Al-Rajabi R . Pemigatinib for previously treated, locally advanced or metastatic cholangiocarcinoma: A multicentre, open-label, phase 2 study. Lancet Oncol. (2020) 21:671–84. doi: 10.1016/S1470-2045(20)30109-1 32203698 PMC8461541

[25] Goyal L Meric-Bernstam F Hollebecque A Valle JW Morizane C Karasic TB . Futibatinib for FGFR2-rearranged intrahepatic cholangiocarcinoma. N Engl J Med. (2023) 388:228–39. doi: 10.1056/NEJMoa2206834 36652354

[26] Subbiah V Lassen U Élez E Italiano A Curigliano G Javle M . Dabrafenib plus trametinib in patients with BRAFV600E-mutated biliary tract cancer (ROAR): A phase 2, open-label, single-arm, multicentre basket trial. Lancet Oncol. (2020) 21:1234–43. doi: 10.1016/S1470-2045(20)30321-1 32818466

[27] Sanchez-Vega F Mina M Armenia J Chatila WK Luna A La KC . Oncogenic signaling pathways in The Cancer Genome Atlas. Cell. (2018) 173:321–337.e10. doi: 10.1016/j.cell.2018.03.035 29625050 PMC6070353

[28] Bailey MH Tokheim C Porta-Pardo E Sengupta S Bertrand D Weerasinghe A . Comprehensive characterization of cancer driver genes and mutations. Cell. (2018) 173:371–385.e18. doi: 10.1016/j.cell.2018.02.060 29625053 PMC6029450

[29] Dong L Lu D Chen R Lin Y Zhu H Zhang Z . Proteogenomic characterization identifies clinically relevant subgroups of intrahepatic cholangiocarcinoma. Cancer Cell. (2022) 40:70–87.e15. doi: 10.1016/j.ccell.2021.12.006 34971568

[30] Ma L Wang L Khatib SA Chang CW Heinrich S Dominguez DA . Single-cell atlas of tumor cell evolution in response to therapy in hepatocellular carcinoma and intrahepatic cholangiocarcinoma. J Hepatol. (2021) 75:1397–408. doi: 10.1016/j.jhep.2021.06.028 34216724 PMC8604764

[31] Ma L Heinrich S Wang L Keggenhoff FL Khatib S Forgues M . Multiregional single-cell dissection of tumor and immune cells reveals stable lock-and-key features in liver cancer. Nat Commun. (2022) 13:7533. doi: 10.1038/s41467-022-35291-5 36476645 PMC9729309

[32] Zhang M Yang H Wan L Wang Z Wang H Ge C . Single-cell transcriptomic architecture and intercellular crosstalk of human intrahepatic cholangiocarcinoma. J Hepatol. (2020) 73:1118–30. doi: 10.1016/j.jhep.2020.05.039 32505533

[33] Affo S Nair A Brundu F Ravichandra A Bhattacharjee S Matsuda M . Promotion of cholangiocarcinoma growth by diverse cancer-associated fibroblast subpopulations. Cancer Cell. (2021) 39:866–882.e11. doi: 10.1016/j.ccell.2021.03.012 33930309 PMC8241235

[34] Zhou PY Zhou C Gan W Tang Z Sun BY Huang JL . Single-cell and spatial architecture of primary liver cancer. Commun Biol. (2023) 6:1181. doi: 10.1038/s42003-023-05455-0 37985711 PMC10661180

[35] von Elm E Altman DG Egger M Pocock SJ Gøtzsche PC Vandenbroucke JP . The Strengthening the Reporting of Observational Studies in Epidemiology (STROBE) statement: Guidelines for reporting observational studies. Lancet (London England). (2007) 370:1453–7. doi: 10.1016/S0140-6736(07)61602-X 18064739

[36] Glickman ME Rao SR Schultz MR . False discovery rate control is a recommended alternative to Bonferroni-type adjustments in health studies. J Clin Epidemiol. (2014) 67:850–7. doi: 10.1016/j.jclinepi.2014.03.012 24831050

[37] Cerami E Gao J Dogrusoz U Gross BE Sumer SO Aksoy BA . The cBio cancer genomics portal: An open platform for exploring multidimensional cancer genomics data. Cancer Discov. (2012) 2:401–4. doi: 10.1158/2159-8290.CD-12-0095 22588877 PMC3956037

[38] Gao J Aksoy BA Dogrusoz U Dresdner G Gross B Sumer SO . Integrative analysis of complex cancer genomics and clinical profiles using the cBioPortal. Sci Signaling. (2013) 6:pl1. doi: 10.1126/scisignal.2004088 23550210 PMC4160307

[39] Boerner T Drill E Pak LM Nguyen B Sigel CS Doussot A . Genetic determinants of outcome in intrahepatic cholangiocarcinoma. Hepatol (Baltimore Md). (2021) 74:1429–44. doi: 10.1002/hep.31829 33765338 PMC8713028

[40] Song Y Boerner T Drill E Shin P Kumar S Sigel C . A novel approach to quantify heterogeneity of intrahepatic cholangiocarcinoma: The hidden-genome classifier. Clin Cancer Res: Off J Am Assoc For Cancer Res. (2024) 30:3499–511. doi: 10.1158/1078-0432.CCR-24-0657 38864854 PMC11326964

[41] Cheng DT Mitchell TN Zehir A Shah RH Benayed R Syed A . Memorial Sloan Kettering-integrated mutation profiling of actionable cancer targets (MSK-IMPACT): A hybridization capture-based next-generation sequencing clinical assay for solid tumor molecular oncology. J Mol Diagn: JMD. (2015) 17:251–64. doi: 10.1016/j.jmoldx.2014.12.006 25801821 PMC5808190

[42] Zehir A Benayed R Shah RH Syed A Middha S Kim HR . Mutational landscape of metastatic cancer revealed from prospective clinical sequencing of 10,000 patients. Nat Med. (2017) 23:703–13. doi: 10.1038/nm.4333 28481359 PMC5461196

[43] Zhang X Bai Q Wang Y Jiang Z Han J Xue C . FGFR2 fusion/rearrangement analysis in intrahepatic cholangiocarcinoma using DNA/RNA-based NGS and FISH. Virchows Arch. (2025) 487:1103–15. doi: 10.1007/s00428-025-04067-9 40198372 PMC12647265

[44] Schubert M Klinger B Klünemann M Sieber A Uhlitz F Sauer S . Perturbation-response genes reveal signaling footprints in cancer gene expression. Nat Commun. (2018) 9:20. doi: 10.1038/s41467-017-02391-6 29295995 PMC5750219

[45] Ma L Hernandez MO Zhao Y Mehta M Tran B Kelly M . Tumor cell biodiversity drives microenvironmental reprogramming in liver cancer. Cancer Cell. (2019) 36:418–430.e6. doi: 10.1016/j.ccell.2019.08.007 31588021 PMC6801104

[46] Robinson MD McCarthy DJ Smyth GK . edgeR: A Bioconductor package for differential expression analysis of digital gene expression data. Bioinf (Oxford England). (2010) 26:139–40. doi: 10.1093/bioinformatics/btp616 19910308 PMC2796818

[47] Robinson MD Oshlack A . A scaling normalization method for differential expression analysis of RNA-seq data. Genome Biol. (2010) 11:R25. doi: 10.1186/gb-2010-11-3-r25 20196867 PMC2864565

[48] McCarthy DJ Chen Y Smyth GK . Differential expression analysis of multifactor RNA-Seq experiments with respect to biological variation. Nucleic Acids Res. (2012) 40:4288–97. doi: 10.1093/nar/gks042 22287627 PMC3378882

[49] Crowell HL Soneson C Germain PL Calini D Collin L Raposo C . muscat detects subpopulation-specific state transitions from multi-sample multi-condition single-cell transcriptomics data. Nat Commun. (2020) 11:6077. doi: 10.1038/s41467-020-19894-4 33257685 PMC7705760

[50] Zimmerman KD Espeland MA Langefeld CD . A practical solution to pseudoreplication bias in single-cell studies. Nat Commun. (2021) 12:738. doi: 10.1038/s41467-021-21038-1 33531494 PMC7854630

[51] Squair JW Gautier M Kathe C Anderson MA James ND Hutson TH . Confronting false discoveries in single-cell differential expression. Nat Commun. (2021) 12:5692. doi: 10.1038/s41467-021-25960-2 34584091 PMC8479118

[52] Holland CH Tanevski J Perales-Patón J Gleixner J Kumar MP Mereu E . Robustness and applicability of transcription factor and pathway analysis tools on single-cell RNA-seq data. Genome Biol. (2020) 21:36. doi: 10.1186/s13059-020-1949-z 32051003 PMC7017576

[53] Jin S Guerrero-Juarez CF Zhang L Chang I Ramos R Kuan CH . Inference and analysis of cell-cell communication using CellChat. Nat Commun. (2021) 12:1088. doi: 10.1038/s41467-021-21246-9 33597522 PMC7889871

[54] Efremova M Vento-Tormo M Teichmann SA Vento-Tormo R . CellPhoneDB: inferring cell-cell communication from combined expression of multi-subunit ligand-receptor complexes. Nat Protoc. (2020) 15:1484–506. doi: 10.1038/s41596-020-0292-x 32103204

[55] Dimitrov D Türei D Garrido-Rodriguez M Burmedi PL Nagai JS Boys C . Comparison of methods and resources for cell-cell communication inference from single-cell RNA-Seq data. Nat Commun. (2022) 13:3224. doi: 10.1038/s41467-022-30755-0 35680885 PMC9184522

[56] World Medical Association . World Medical Association Declaration of Helsinki. Ethical principles for medical research involving human subjects. Nurs Ethics. (2002) 9:105–9. doi: 10.1191/0969733002ne486xx 16010903

[57] Cadamuro M Nardo G Indraccolo S Dall'olmo L Sambado L Moserle L . Platelet-derived growth factor-D and Rho GTPases regulate recruitment of cancer-associated fibroblasts in cholangiocarcinoma. Hepatol (Baltimore Md). (2013) 58:1042–53. doi: 10.1002/hep.26384 23505219 PMC3732815

[58] Cadamuro M Brivio S Mertens J Vismara M Moncsek A Milani C . Platelet-derived growth factor-D enables liver myofibroblasts to promote tumor lymphangiogenesis in cholangiocarcinoma. J Hepatol. (2019) 70:700–9. doi: 10.1016/j.jhep.2018.12.004 30553841 PMC10878126

[59] Vaquero J Lobe C Tahraoui S Clapéron A Mergey M Merabtene F . The IGF2/IR/IGF1R pathway in tumor cells and myofibroblasts mediates resistance to EGFR inhibition in cholangiocarcinoma. Clin Cancer Res: Off J Am Assoc For Cancer Res. (2018) 24:4282–96. doi: 10.1158/1078-0432.CCR-17-3725 29716918

