# Supplementary material for: RTK–RAS pathway alterations in hepatocellular carcinoma and cholangiocarcinoma: a multicohort genomic and single-cell transcriptomic study
Source: Front Oncol. 2026 Oct 1;16:1963032. doi: 10.3389/fonc.2026.1963032 (PMC13669719; doi:10.3389/fonc.2026.1963032)
Supplement: Supplementary file 1 [file DataSheet1.docx]

Supplementary Figure S1. Sample-level quality control and cell-composition characteristics of the two single-cell RNA-sequencing cohorts

**A**, Numbers of annotated cells in each analyzed sample from GSE189903 (Multi-regional) and GSE151530 (NCI-CLARITY), partitioned into B cells, CAFs, malignant cells, T/NK cells, TAMs, TECs, and unclassified cells. Each stacked bar represents one sample; colors indicate cell annotation.

**B,** Sample-level malignant-cell fractions in HCC and iCCA, shown separately for the two datasets. Boxes indicate the median and interquartile range, whiskers extend to 1.5 times the interquartile range, and points represent samples. The display is descriptive; patient-level inference used within-patient aggregation as specified in the Methods.

**C,** Sequencing-complexity summary for each sample. The x-axis shows the cell-count-weighted median number of detected genes per cell and the y-axis shows the cell-count-weighted median UMI count per cell. Points are colored by cancer type and panels denote datasets.

**D,** Distribution of sample-level fractions for the six resolved cell compartments in each dataset. Boxes indicate the median and interquartile range; whiskers extend to 1.5 times the interquartile range. B, B cells; CAF, cancer-associated fibroblasts; TAM, tumor-associated macrophages; TEC, tumor endothelial cells.
